# The trends in the incidence and thrombosis-related comorbidities of antiphospholipid syndrome: a 14-year nationwide population-based study

**DOI:** 10.1186/s12959-022-00409-8

**Published:** 2022-09-01

**Authors:** Wei-Cheng Yao, Kam-Hang Leong, Lu-Ting Chiu, Po-Yi Chou, Li-Chih Wu, Chih-Yu Chou, Chien-Feng Kuo, Shin-Yi Tsai

**Affiliations:** 1 Department of Anesthesiology and Pain Medicine, Min-Sheng General Hospital, Tao-Yuan City, Taiwan; 2 Department of Laboratory Medicine, MacKay Memorial Hospital, No. 92, Sec. 2, Zhongshan N. Rd, Taipei City, 10449 Taiwan; 3 Department of Medicine, MacKay Medical College, New Taipei City, Taiwan; 4 Management Office for Health Data, China Medical University Hospital, Taichung City, Taiwan; 5 Division of Infectious Diseases, Department of Internal Medicine, MacKay Memorial Hospital, Taipei, Taiwan; 6 Department of Nursing, MacKay Junior College of Medicine, Nursing and Management, New Taipei City, Taiwan; 7 Institute of Biomedical Sciences, MacKay Medical College, New Taipei City, Taiwan; 8 Institute of Long-Term Care, MacKay Medical College, New Taipei City, Taiwan; 9 Department of Health Policy and Management, Johns Hopkins Bloomberg School of Public Health, Johns Hopkins University, Baltimore, MD USA

**Keywords:** Antiphospholipid syndrome, Epidemiology, Incidence, National health programs, Nationwide population-based study

## Abstract

**Background:**

This study aims to provide 14-year nationwide epidemiology data to evaluate the incidence ratio of APS in Taiwan and the condition of comorbidities by analyzing the National Health Insurance Research Database.

**Methods:**

Nineteen thousand one hundred sixty-three patients newly diagnosed as having APS during the 2000–2013 period and 76,652 controls (with similar distributions of age and sex) were analyzed.

**Results:**

The incidence of APS increased from 4.87 to 6.49 per 10,000 person-years in the Taiwan population during 2000–2013. The incidence of APS increased with age after 20 years old, especially in the female population, and it rose rapidly after age over 60 years old. In addition, APS cohorts presented a higher proportion of diabetes mellitus, hypertension, hyperlipidemia, stroke, heart failure, atrial fibrillation, myocardial infarction, PAOD, chronic kidney disease, COPD, deep vein thrombosis, pulmonary embolism, SLE, rheumatoid arthritis, Sjogren’s syndrome, and polymyositis.

**Conclusions:**

Our study indicated an increasing trend in APS incidence among the Taiwanese population and a relationship between APS and potential comorbidities. This large national study found that the APS risk is heavily influenced by sex and age. Thus, the distinctive sex and age patterns might be constructive given exploring potential causal mechanisms. Furthermore, our findings indicate that clinicians should have a heightened awareness of the probability of APS, especially in women in certain age groups presenting with symptoms of APS.

## Introduction

Antiphospholipid syndrome (APS) is an autoimmune disease associated with the presence of antiphospholipid antibodies(aPLs), such as anticardiolipin antibodies, antiβ2-glycoprotein 1 antibodies, and lupus anticoagulant. APS diagnosis is based on the combination of clinical features, including thrombosis in the arteries veins, or small-vessels and/or obstetrical complications such as recurrent miscarriage and the detection of circulating aPLs [1]. However, the pathophysiology of APS remains largely unknown. Several mechanisms have been proposed, including the binding of aPLs to β2-glycoprotein 1 receptors, endothelial cell dysfunction, low activity of the epithelial nitric oxide system (eNOS), and complement activation and disposition. APS eventually leads to obstetric or thrombotic complications [2].

Because APS is a rare disease, high-quality epidemiological data from various ethnicities or groups with different comorbidities are required. Recently, a population-based study of 144,248 participants reported that during a 16-year study period, 33 incident cases were recorded, and the annual incidence and estimated prevalence of APS were approximately two people per 10^5^ person-years and 50 per 10^5^ people, respectively [3]. Another study based on the Korean Health Insurance and Review Agency database, which contains data on more than 52 million Koreans, revealed a total of 3088 newly diagnosed incident cases (1215 men and 1873 women) during 2009–2016. The incidence rate was 0.75 per 10^5^ person-years, and the prevalence rate in 2016 was 6.19 per 10^5^ individuals [4].

This study aims to provide ten-year nationwide epidemiology data to evaluate the incidence ratio of APS and the condition of comorbidities, which also affect the risk of thrombosis.

## Method

### Data sources

Taiwan’s National Health Insurance (NHI) program began in 1995 and covered approximately 99% of the 23 million people living in Taiwan [5–8]. This study used the hospitalization dataset from the National Health Insurance Research Database, which contains all inpatient insurance claims filed in Taiwan from 1996 to 2013. The database includes comprehensive information on inpatient care and provides researchers with encrypted personal data associated with relevant claims information, including demographic data, disease diagnosis, and treatments. The disease diagnosis is based on the *International Classification of Diseases, Ninth Revision, Clinical Modification* (ICD-9-CM) codes [9].

### Definition of APS and non-APS cohorts

This study identified all patients with APS who received their diagnosis between January 1, 2000, to December 31, 2013, to calculate the incidence rates in Taiwan. All patients diagnosed as having APS as defined based on 2006 Revised classification criteria for antiphospholipid syndrome [1]. ICD-9-CM codes 286.53, 289.8, 287.3, 795.7, and 649.3 were classified as the case group. The date of the initial diagnosis of APS was set as the index date. For each APS patient, four cohorts without APS were randomly selected from the same database and frequency-matched according to age, sex, and index year at a 1:4 ratio. The index year for the (control) participants without APS was randomly assigned.

### Potential comorbidities

APS-related comorbidities in the study population that were considered included diabetes mellitus [10] (ICD-9-CM 250), hypertension [11–13] (ICD-9-CM 401–405), hyperlipidemia [14] (ICD-9-CM 272), stroke [10](ICD-9-CM 430–438), heart failure [15] (ICD-9-CM 428), atrial fibrillation [16] (ICD-9-CM 427.32), myocardial infarction [17] (ICD-9-CM 410–410.9, 412), peripheral arterial occlusive disease [10] (PAOD; ICD-9-CM 440–444), chronic kidney disease [13] (ICD-9-CM 580–589), chronic obstructive pulmonary disease [18](COPD; ICD-9-CM 490–496), deep vein thrombosis [10] (ICD-9-CM 451.1, 451.2, 451.8, and 453), pulmonary embolism [10] (ICD-9-CM 415.1), systemic lupus erythematosus [10] (SLE; ICD-9-CM 710.0), rheumatoid arthritis [19] (ICD-9-CM 714), systemic sclerosis [20] (ICD-9-CM 710.1), Sjogren’s syndrome [21] (ICD-9-CM 710.2), and polymyositis [22] (ICD-9-CM 710.4).

### Statistical analysis

We calculated the annual incidence and age-specific incidence during the 2000–2013 period. The annual incidence was defined as the number of patients with APS divided by the total person-years (per 10,000 person-years) of people in the NHI program annually [23]. The age-specific incidence of APS was calculated by dividing the total person-years (per 10,000 person-years) in each age group (10-year intervals). The ages of the study population were defined as their age in the middle of the follow-up period. We further stratified incidence by sex subgroups. Poisson regression was used to analyze trends of incidence by index year and in each age group. The descriptive statistics of the participants with APS and those without APS were summarized as means and standard deviations for continuous variables; data were presented as cases and percentages for categorical variables. Differences in age group, sex, and comorbidities between participants with and without APS were examined using the chi-square test; age distributions were analyzed using the independent-samples t-test. All statistical analyses were conducted using the SAS package (version 9.4; SAS Institute Inc., Cary, NC, United States). The incidence curve was generated using R software (R Foundation for Statistical Computing, Vienna, Austria). A two-tailed *p*-value less than 0.05 was considered statistically significant.

### Statement

According to previously established study designs, we conducted this study using data from the LHID. The present study was an analysis of de-identified and encrypted secondary data; therefore, no informed consent was required. This study was approved by the Institutional Review Board of China Medical University (CMUH-104-REC2–115(CR-6)), Min-Sheng General Hospital (NO:2022001) and MacKay Memorial Hospital (16MMHIS074). We confirmed that all the methods in this research were performed in accordance with the relevant guidelines and regulations.

## Result

**Fig. 1:**
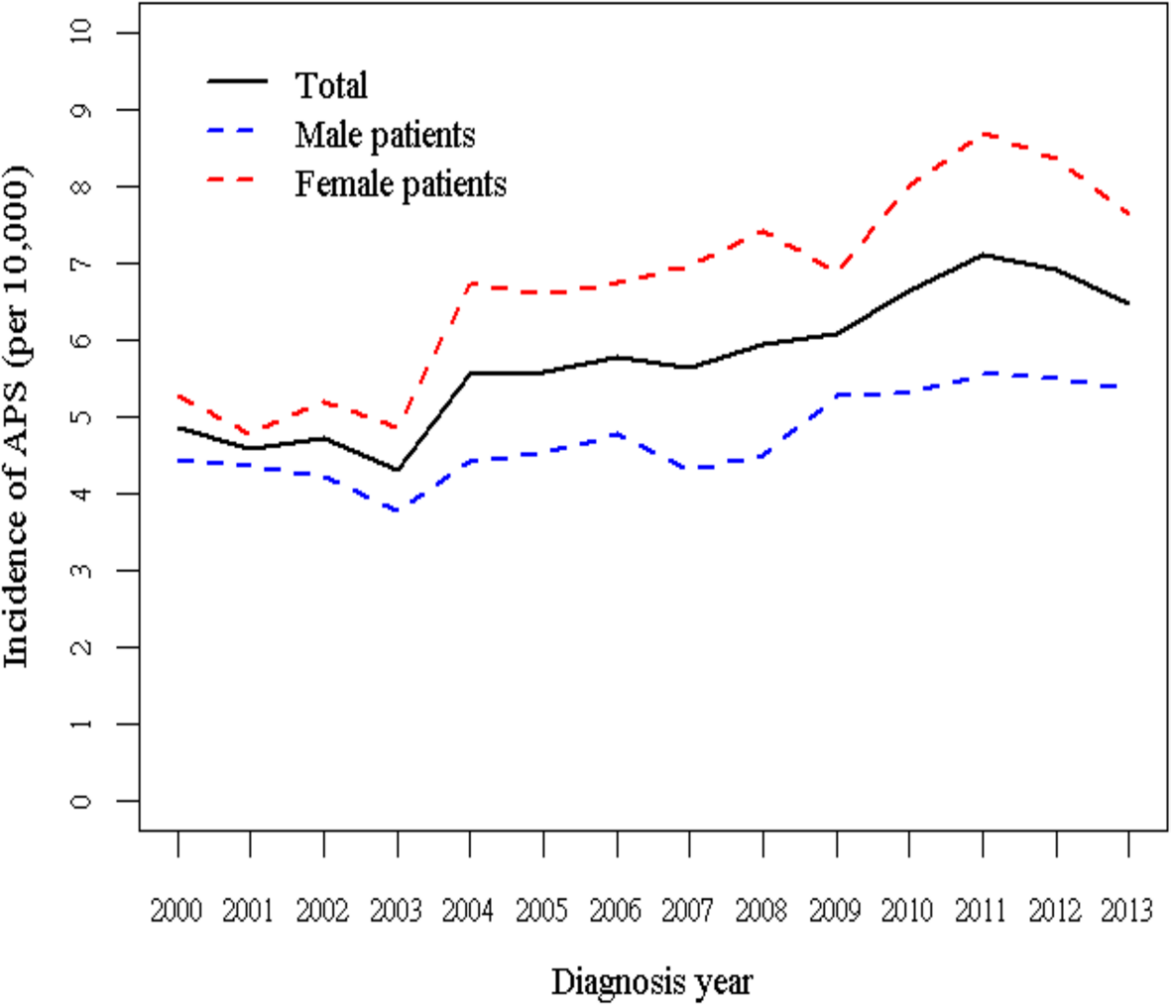
The annual incidence rate of APS during 2000–2013 in Taiwan

Table 1 and Fig. 1 display the annual incidence rates of APS for male and female participants separately during the 2000–2013 period. The study population included 19,163 patients newly diagnosed as having APS, of whom 7926 (41.36%) were male patients, and 11,237 (58.64%) were female patients (Table 2). The incidence of APS increased from 4.87 to 6.49 per 10,000 person-years in the total population during 2000–2013. With respect to sex subgroups, the incidence increased from 4.44 to 5.36 per 10,000 person-years in the male population and from 5.27 to 7.62 per 10,000 person-years in the female population. Overall, women exhibited a higher annual APS incidence than men.

**Table 1:** Annual incidence of Antiphospholipid Syndrome stratified by gender

Index year	Total			Male			Female		
	cases	person-year	incidence rate (95% CI)	cases	person-year	incidence rate	cases	person-year	incidence rate
2000	1111	22,823,451	4.87	497	11,181,321	4.44	614	11,642,130	5.27
2001	1060	23,170,145	4.57	497	11,408,052	4.36	563	11,762,093	4.79
2002	1102	23,365,986	4.72	487	11,550,720	4.22	615	11,815,266	5.21
2003	1014	23,490,871	4.32	439	11,648,599	3.77	575	11,842,272	4.86
2004	1316	23,642,983	5.57	519	11,767,614	4.41	800	11,875,369	6.74
2005	1328	23,785,305	5.58	539	11,875,098	4.54	789	11,910,207	6.62
2006	1378	23,892,455	5.77	572	11,953,923	4.79	806	11,938,532	6.75
2007	1353	23,999,660	5.64	517	12,035,496	4.30	836	11,964,164	6.99
2008	1435	24,099,774	5.95	546	12,112,765	4.51	889	11,987,009	7.42
2009	1466	24,140,628	6.07	641	12,164,240	5.27	825	11,976,388	6.89
2010	1608	24,193,125	6.65	649	12,219,631	5.31	959	11,973,494	8.01
2011	1725	24,289,377	7.10	681	12,281,805	5.54	1044	12,007,572	8.69
2012	1687	24,373,355	6.92	680	12,348,656	5.51	1007	12,024,699	8.37
2013	1580	24,354,771	6.49	662	12,352,693	5.36	915	12,002,078	7.62
P for trend			<.0001			<.0001			<.0001

Incidence rate, per 10,000 person-years

**Table 2:** Characteristics among patients with Antiphospholipid Syndrome

	Antiphospholipid Syndrome (***n*** = 19,163)	
Characteristics	n	%
**Age, years**		
0–10	3242	16.92
11–20	1112	5.80
21–30	1964	10.25
31–40	2709	14.14
41–50	2021	10.55
51–60	2150	11.22
61–70	2031	10.60
71–80	2368	12.36
> 80	1566	8.16
mean ± SD	43.02 ± 26.45	
**Gender**		
Female	11,237	58.64
Male	7926	41.36
**Comorbidity**		
Diabetes mellitus	3548	18.51
Hypertension	3632	18.95
Hyperlipidemia	2729	14.24
Stroke	4006	20.90
Heart failure	2646	13.81
Atrial fibrillation	411	2.15
Myocardial infarction	2197	11.46
PAOD	1879	9.81
Chronic kidney disease	3072	16.03
COPD	3672	19.16
Deep vein thrombosis	1167	6.09
Pulmonary embolism	410	2.14
Systemic lupus erythematosus	419	2.19
Rheumatoid arthritis	816	4.26
Systemic sclerosis	53	0.28
Sjogren’s syndrome	498	2.6
Polymyositis & dermatomyositis	34	0.18

Data shown as n(%) or mean ± SD

**Fig. 2:**
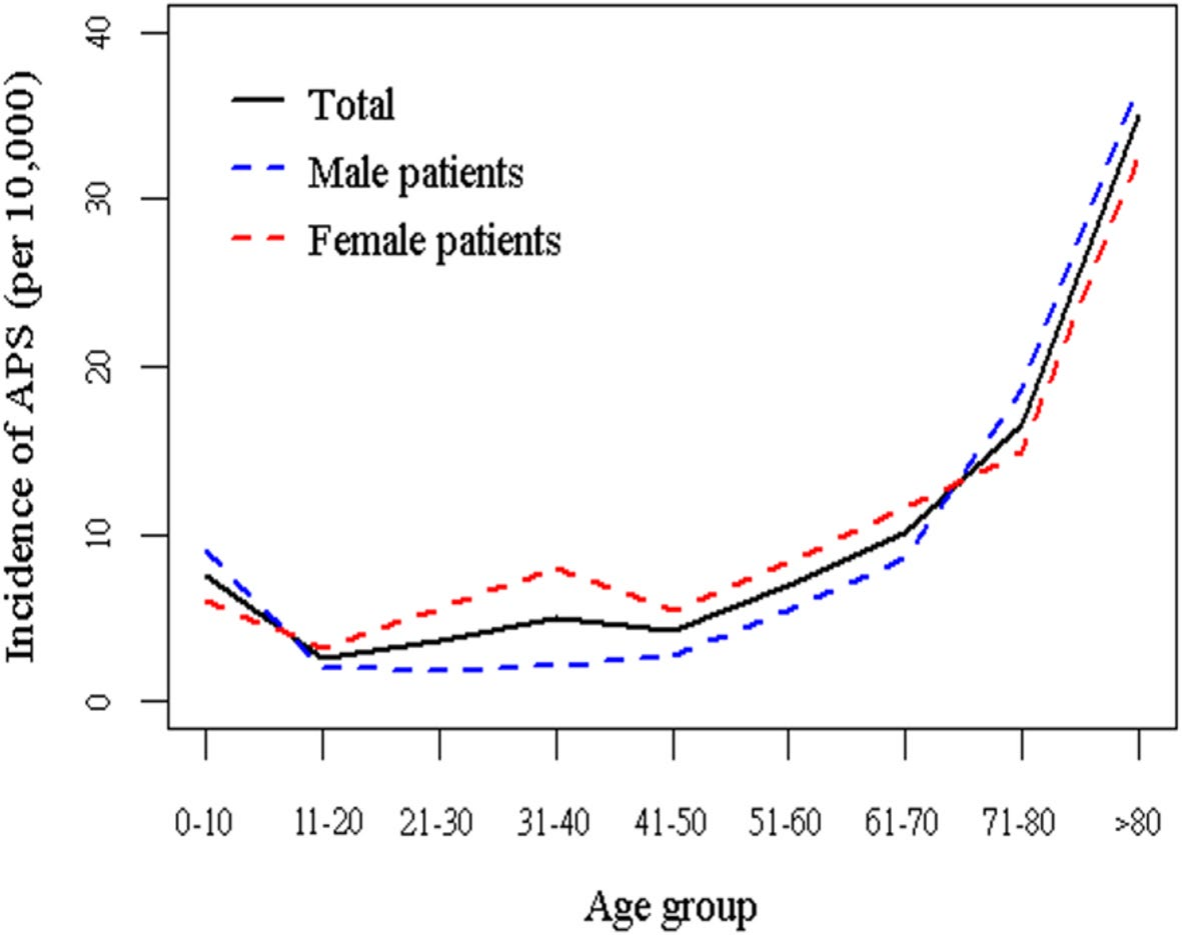
The incidence of APS cases by sex among age groups

The age-specific incidences of APS are presented in Table 3 and Fig. 2. Compared with the 0–10 age group, cases in the 11–20 age group markedly decreased from 7.52 per 10,000 person-years in the 0–10 age group to 2.67 per 10,000 person-years in the 11–20 age group. After the age of 20, the incidence of APS increased with age and rose rapidly after age 60 years in both the male and female populations. The female population exhibited a higher incidence of APS in the 11–80 age group, whereas the male population recorded a higher incidence of APS in the 0–10 and > 80 age groups.

**Table 3:** Incidence of Antiphospholipid Syndrome stratified by gender among each age group

Age	Total			Male			Female		
	cases	person-year	incidence rate	cases	person-year	incidence rate	cases	person-year	incidence rate
0–10	3242	43,104,064	7.52	1879	20,597,858	9.12	1363	22,506,206	6.06
11–20	1112	41,682,562	2.67	411	19,988,749	2.06	701	21,693,813	3.23
21–30	1964	53,879,919	3.65	516	27,818,813	1.85	1448	26,061,106	5.56
31–40	2709	53,481,640	5.07	589	27,105,783	2.17	2120	26,375,857	8.04
41–50	2021	48,388,269	4.18	674	23,992,840	2.81	1347	24,395,429	5.52
51–60	2150	31,268,709	6.88	848	15,630,505	5.43	1302	15,638,204	8.33
61–70	2031	20,243,970	10.03	887	10,329,812	8.59	1144	9,914,158	11.54
71–80	2368	14,267,786	16.60	1241	6,668,043	18.61	1127	7,599,743	14.83
> 80	1566	4,481,516	34.94	881	2,382,772	36.97	685	2,098,744	32.64
P for trend			<.0001			<.0001			<.0001

Incidence rate, per 10,000 person-years

The results of the Poisson regression model revealed that age, sex, and index year were significantly associated with the incidence of APS (*p* < 0.0001). It suggests that the annual incidence of APS increased markedly with increasing age and predominantly in the female population.

Table 4 details the characteristics of age, sex, and comorbidities of the participants with and without APS. After frequency matching, 19,163 patients with APS and 76,652 controls (with similar distributions of age and sex) were analyzed. The mean ages of the participants with APS and those without APS were 43.02 ± 26.45 years and 42.71 ± 26.21 years, respectively (*p* = 0.49). Thus, more than 40% of cases were aged over 40 years. Compared with the participants without APS, more participants with APS also had cardiovascular risk factors (diabetes mellitus, hypertension, hyperlipidemia, stroke, heart failure, atrial fibrillation and myocardial infarction), autoimmune associations (SLE, rheumatoid arthritis, Sjogren’s syndrome, and polymyositis), chronic kidney disease and COPD. the APS group most presented with the comorbidities of PAOD, deep vein thrombosis, pulmonary embolism, (*p* < 0.001).

**Table 4:** Characteristics among patients with Antiphospholipid Syndrome

Characteristics	Antiphospholipid Syndrome				***p***-value
	No (***n*** = 76,652)		Yes (***n*** = 19,163)		
	n	%	n	%	
**Age, years**					0.49
< 40	36,441	47.54	9027	47.11	
40–60	16,662	21.74	4171	21.77	
> 60	23,549	30.72	5965	31.13	
mean ± SD	42.71 ± 26.21		43.02 ± 26.45		0.15
**Gender**					0.82
Female	45,015	58.73	11,237	58.64	
Male	31,637	41.27	7926	41.36	
**Comorbidity**					
Diabetes mellitus	323	0.42	3548	18.51	<.0001
Hypertension	308	0.40	3632	18.95	<.0001
Hyperlipidemia	267	0.35	2729	14.24	<.0001
Stroke	285	0.37	4006	20.90	<.0001
Heart failure	302	0.39	2646	13.81	<.0001
Atrial fibrillation	313	0.41	411	2.14	<.0001
Myocardial infarction	284	0.37	2197	11.46	<.0001
PAOD	256	0.33	1879	9.81	<.0001
Chronic kidney disease	324	0.42	3072	16.03	<.0001
COPD	348	0.45	3672	19.16	<.0001
Deep vein thrombosis	238	0.31	1167	6.09	<.0001
Pulmonary embolism	284	0.37	410	2.14	<.0001
Systemic lupus erythematosus	319	0.42	419	2.19	<.0001
Rheumatoid arthritis	305	0.40	816	4.26	<.0001
Systemic sclerosis	284	0.37	53	0.28	0.03
Sjogren’s syndrome	26	0.03	498	2.6	<.0001
Polymyositis & dermatomyositis	9	0.01	34	0.18	<.0001

Data shown as n(%) or mean ± SD

Using 1:4 frequency matching

## Discussion

This is the most extensive study of APS incidence to date, including 19,163 participants with APS and using national population-based registry data. To our knowledge, this is also the first study to investigate the distributions of APS by sex and age in a population. Most epidemiological studies have not reported age distributions due to small study populations or have only declared the age distribution for women and men combined. Our study found that the female population had a higher annual incidence rate of APS than the male population. Female-to-male ratios ranging from 5:1 to 2:1 were reported in one study [24]. Furthermore, an increasing trend in APS incidence among the Taiwanese population was observed in our study. Environmental factors influence the onset of autoimmune diseases. However, an earlier study indicated that infection and drug exposure were correlated with APS [25]. The venereal disease research laboratory test, which involves using purified cardiolipin–lecithin–cholesterol antigen to detect anticardiolipin antibodies, is a screening test for syphilis; it can also positively identify autoimmune diseases such as APS and SLE [26]. Exposure to various bacteria and viruses, including *Mycoplasma pneumonia, Streptococcus pyogenes*, *Helicobacter pylori*, Epstein–Barr virus, and cytomegalovirus, is associated with an increased prevalence of aPLs [25]. Several drugs are involved in autoimmunity, including those that produce drug-induced lupus and drug-induced autoimmune hepatitis. Specifically, certain medications, such as procainamide, chlorothiazide, phenothiazines, quinine, and oral contraceptives, are associated with increased levels of aPLs [27]. Future studies are necessary to determine correlations between APS and environmental factors.

APS is characterized by vascular thromboses and pregnancy-related morbidity associated with persistently elevated aPLs [1], which are autoantibodies that recognize a variety of phospholipid-binding plasma proteins beta2-glycoprotein I, prothrombin, and annexin A5. The main pathogenetic mechanisms of aPL-induced thrombosis involve stimulation of the extrinsic coagulation pathway, platelet aggregation, and complement activation and inhibition of tPA, protein C, and protein S [28]. Oxidative stress was reported to affect the structure and function of beta2-glycoprotein I, a complement control protein constructed of five domains [29]. There are two forms of beta2-glycoprotein I – free thiol form (contains broken disulfide bridge at cysteine (Cys) 32 and Cys 60 in domain I and Cys 288 and Cys 326 in domain V) and oxidized form (contains disulfide bonds at these sites) [30]. The level of oxidized form was significantly higher in patients with APS. Lower levels of free thiol form cause a lack of buffer against oxidative stress [31]. Oxidative stress from exogenous sources followed by vascular endothelial injury can stimulate platelet aggregation and von Willebrand factor expression [32]. Antibodies binding to a particular epitope in domain I of beta2-glycoprotein I have been indicated to increase the risk of thrombosis [33]. Furthermore, beta2-glycoprotein I immune complexes can induce up-regulated activation of toll-like receptor 7 (TLR7) in plasmacytoid dendritic cells and monocytes to release pro-inflammatory cytokine and create a positive-feedback loop for further autoantibody generation [34].. Understanding these pathophysiologies provide insight into APS management. Rituximab, a chimeric monoclonal antibody that targets CD20, inhibits B cells involved in aPL-induced clinical manifestations of APS [35]. Hydroxychloroquine has been reported to decrease the overexpression of GPIIbIIIa on the membrane of aPL-activated platelets and inhibit platelet aggregation [36]. In pregnancy-related morbidity, 20% of female patients with APS experience recurrent pregnancy losses, including miscarriage, fetal loss, and stillbirth at any stage of pregnancy [37]. aPL binding to monocytes, endothelial cells, platelets, and plasma components of the coagulation cascade in the induction of thrombosis causes fetal death in APS. Direct effects of anti-β2GPI autoantibodies on the placenta include an inflammatory response resulting in trophoblast damage, binding to cultured cytotrophoblast cells that causes trophoblast membrane perturbation, and a reduction in the secretion of human chorionic gonadotropin [38, 39].

Our study discovered that patients with APS suffered more comorbidities such as hypertension, hyperlipidemia, heart failure, atrial fibrillation, and chronic kidney disease. To the best of our knowledge, one study indicated that the patients with hypertension have higher IgG levels of antibodies to endothelial cells and β2GPI (Beta-2-Glycoprotein I) than control groups. Furthermore, elevated insulin levels, insulin-like growth factor binding protein-1, and greater insulin resistance were associated with Anti-β2GP1 levels. These findings were correlated to our result and provided evidence of linkage between APS and metabolic variables [40]. Adipocytokine, a product produced by adipose tissues, was believed to contribute to low-grade inflammation and several diseases such as metabolic syndrome, atherosclerosis, and type 2 diabetes mellitus [41]. The patients with primary APS and coexistence of metabolic syndrome were reported to have more risks of arterial events by the deterioration of existing endothelial cell dysfunction [42].

Since the initial descriptions of APS were developed, hypertension has been considered one of the frequent signs related to the disease. Hughes identified that an association between livedo reticularis and elevated blood pressure contributed to renovascular etiology; the study population included patients with APS with varying degrees of hypertension ranging from mildly elevated to malignant [11, 12]. Renal involvement was an etiology of the elevated blood pressure in APS [13]. One research demonstrated an extensive series of renal biopsies in APS patients with renal manifestation. Vascular nephropathies such as small vessel vaso-occlusive lesions, recanalizing thrombi in arteries and arterioles, and focal cortical atrophy were found. In addition, 93% of those participants had systemic hypertension; given the high prevalence of hypertension in APS nephropathy (APSN), elevated blood pressure is considered a key marker of renal status [43]. One study indicated that anti-prothrombin antibodies are related to hypertension through a comparison of a patient group with severe essential hypertension with a matched group of healthy controls; it revealed that 8% of the participants in a patient group had anti-prothrombin antibodies compared with none of the healthy controls [44]. Shajit Sadanand et al. mentioned the association between lipid profile and aPLs. The most general dyslipidemia case in the study population is TG level > 150 mg/dL(51.9%), while LDL > 150 mg/dL(40.2%) takes second place. Statistics show a significant correlation among anti-β2G IgG levels, HDL and LDL level, and aCL IgM level and LDL [14]. Antibodies to oxidized LDLs and cardiolipins were associated with thrombosis and atherosclerotic complications in patients with SLE as early as 1993 [45]. These antibodies interfere with the regulation between platelets, endothelial cells, and coagulation factors and disturb the balance of coagulation and Fibrinolysis [46, 47]. One study suggested the assay of anti-β2GP1 with lupus anticoagulant can be used for early detection to those with APS and e thromboembolic events [48].

Our study population was mainly composed of East Asians living in Taiwan. We confirmed that APS was strongly associated with other autoimmune diseases, a finding that is consistent with research undertaken in Western countries. In one such cohort study, up to 36% of patients with APS were observed to have a history of SLE [10]. Compared with patients diagnosed as having primary APS, patients with APS as well as an SLE history presented increased incidences of arthralgias and arthritis, leukopenia, autoimmune hemolytic anemia, livedo reticularis, epilepsy, and myocardial infarction [17]. Furthermore, those patients exhibited higher rates of hypertension, dyslipidemia, diabetes, and severe lupus profiles with major organ involvement and higher rates of mortality [10, 49]. Their conditions required long-term anticoagulant treatment and immunosuppressive therapy, including high-dose corticosteroids, cyclophosphamide, and azathioprine [50].

The strength of this study was its employment of a database containing nationwide population-based data of approximately 99% of the 23 million people living in Taiwan. The database’s reliability and validity for epidemiological investigations have been reported previously [51]. The use of the ICD-10 definition of APS is uncommon rather than the more standard ICD-9-CM one. However, we suggest that future studies seek access to the medical records and laboratory data to investigate the diagnostic criteria applied to individual medical examinations. The limitation of this study was the anonymity of the NHIRD. The patients’ personal information, family histories and laboratory data were not available.

Our current findings indicate a relationship between APS and nonautoimmune comorbidities, such as hypertension, hyperlipidemia, heart failure, atrial fibrillation, and chronic kidney disease.

## Conclusion

In summary, in addition to the known pathophysiology of the disease, various thrombosis-related diseases may influence the risk for APS. Knowing how APS is distributed by sex and age in a population aid in understanding the relationship between APS and complications. Further research is warranted to investigate the relationship between nonautoimmune comorbidities and APS. Additionally, this study underscores how clinicians should pay close attention to APS-related complications, especially in patient groups prone to high incidences of such complications.

## Data Availability

The data underlying this study is from the National Health Insurance Research Database (NHIRD). Interested researchers can obtain the data through formal application to the Ministry of Health and Welfare, Taiwan.

## Abbreviations

APS: Antiphospholipid syndrome
aPLs: Antiphospholipid antibodies
NHI: National Health Insurance
PAOD: Peripheral arterial occlusive disease
COPD: Chronic obstructive pulmonary disease
SLE: Systemic lupus erythematosus
IgG: Immunoglobulin G
IgM: Immunoglobulin M
APSN: APS nephropathy
β2GPI: Beta-2-Glycoprotein I
